# Efficient Preparation of Enantiopure D-Phenylalanine through Asymmetric Resolution Using Immobilized Phenylalanine Ammonia-Lyase from *Rhodotorula glutinis* JN-1 in a Recirculating Packed-Bed Reactor

**DOI:** 10.1371/journal.pone.0108586

**Published:** 2014-09-30

**Authors:** Longbao Zhu, Li Zhou, Nan Huang, Wenjing Cui, Zhongmei Liu, Ke Xiao, Zhemin Zhou

**Affiliations:** 1 Key Laboratory of Industrial Biotechnology, Ministry of Education, School of Biotechnology, Jiangnan University, Wuxi, Jiangsu, China; 2 School of Biochemical Engineering, Anhui Polytechnic University, Wuhu, Anhui, China; University of Wisconsin, Food Research Institute, United States of America

## Abstract

An efficient enzymatic process was developed to produce optically pure D-phenylalanine through asymmetric resolution of the racemic DL-phenylalanine using immobilized phenylalanine ammonia-lyase (*Rg*PAL) from *Rhodotorula glutinis* JN-1. *Rg*PAL was immobilized on a modified mesoporous silica support (MCM-41-NH-GA). The resulting MCM-41-NH-GA-*Rg*PAL showed high activity and stability. The resolution efficiency using MCM-41-NH-GA-*Rg*PAL in a recirculating packed-bed reactor (RPBR) was higher than that in a stirred-tank reactor. Under optimal operational conditions, the volumetric conversion rate of L-phenylalanine and the productivity of D-phenylalanine reached 96.7 mM h^−1^ and 0.32 g L^−1^ h^−1^, respectively. The optical purity (*ee*_D_) of D-phenylalanine exceeded 99%. The RPBR ran continuously for 16 batches, the conversion ratio did not decrease. The reactor was scaled up 25-fold, and the productivity of D-phenylalanine (*ee*_D_>99%) in the scaled-up reactor reached 7.2 g L^−1^ h^−1^. These results suggest that the resolution process is an alternative method to produce highly pure D-phenylalanine.

## Introduction

D-amino acids are used as intermediates for the synthesis of β-lactam antibiotics and other pharmaceuticals [1], [2]. Chemical, fermentative, and enzymatic methods have already been developed to synthesize D-amino acids [3], [4]. Enzymatic methods are most suited for the industrial manufacture of D-amino acids in regard to their optical purity and productivity, and offer an efficient, highly specific and environmentally friendly alternative to chemical and fermentation methods. For the enzymatic methods, the hydantoinase-carbamoylase method is the primary method used for the commercial production of D-amino acids [5], [6]. In this two-step process, the DL-5-monosubstituted hydantoin used as a starting material is hydrolyzed by D-hydantoinase. The resulting N-carbamoyl-D-amino acid is subsequently hydrolyzed by carbamoylase to yield the free D-amino acid stereospecifically. However, the activity of the carbamoylase is much lower than that of D-hydantoinase, and the enzymes are relatively unstable making the process unsuitable for industrial application [7]. Therefore, carbamoylase catalysis is the rate-determining step in the process [6], [8], [9]. Moreover, the starting substrate, DL-5-monosubstituted hydantoin is not widely available and must be produced through the enzymatic racemization of L-5-monosubstituted hydantoin. The racemization rate of the L-5-monosubstituted hydantoins is very low by hydantoin racemase [10], [11]. The operation process is complex because it occurs in a multi-enzyme catalysis system. To establish the industrial manufacture of D-amino acids, the availability of feedstock, the optical purity of products and the enzymatic reaction steps should be considered. From an industrial perspective, the availability of cheap feedstock and the development of enzyme catalysts suitable for feedstock are the most important considerations. The racemic DL-amino acids are commercially produced at low cost by fermentation and chemical synthesis [12]. If these DL-amino acids can be used directly as feedstock, a simple and efficient D-amino acid manufacturing process should be developed to improve the economic benefits [4], [13]. Therefore, the direct enzymatic resolution of DL-racemic mixtures may be an alternative method for the production of highly optically pure D-amino acids.

D-Phenylalanine is an important chiral component of nateglinide, a drug for the treatment of type 2 diabetes, and widely used as intermediates in the synthesis of antibiotics, antiviral, analgesic and antistress agents [14], [15]. The phenylalanine ammonia-lyase (PAL) can stereoselectively catalyze the conversion of L-phenylalanine into *trans*-cinnamic acid and ammonia, and can be used in the chiral resolution of DL-phenylalanine to produce D-phenylalanine. Moreover, the solubility of *trans*-cinnamic acid is low at acidic pH (approximately 0.006 g/L in aqueous solution at 25°C pH 5), and D-phenylalanine can be easily separated from the reaction solution by regulating the pH. Therefore, the asymmetric resolution of racemic DL-phenylalanine by PAL is an attractive method and shows commercial application potential. In our previous work, a strain with higher PAL activity was discovered from soil and identified as *Rhodotorula glutinis* designed JN-1 (CCTCC NO: M2011490). The *Rg*PAL gene from *Rhodotorula glutinis* JN-1was isolated, successfully expressed in *E. coli* and characterized. The specific activity of *Rg*PAL was 4.2 U/mg, and the *k_cat_*/*K_m_* was 1.92×10^4^ mM^−1^s^−1^, demonstrating the highest catalytic activity among the reported PALs [16]. Consequently, we have investigated the production of D-phenylalanine using *Rg*PAL to asymmetrically resolve the racemic DL-phenylalanine.

Here, we describe an enzymatic method for the production of D-phenylalanine using *Rg*PAL. *Rg*PAL was immobilized on MCM-41 through covalent binding, which increased the operational stability. Then, we set up a D-phenylalanine production process in a recirculating packed-bed reactor (RPBR). The conversion ratio of L-phenylalanine and the optical purity (*ee*_D_) of D-phenylalanine both exceeded 99%, and the RPBR showed high stability even after running continuously for 16 cycles and a total of 384 hours. The reactor was scaled up 25-fold, and the productivity of D-phenylalanine (*ee*_D_>99%) in the scaled-up reactor reached 7.2 g L^−1^h^−1^. The performance of the immobilized PAL was evaluated and the process was effective in producing high-purity D-phenylalanine.

## Material and Methods

### Material

3-Aminopropyltriethoxysilane (98%, APTES), glutaraldehyde (GA), D-phenylalanine and L-phenylalanine were purchased from Sigma (USA). The support of immobilization (MCF-41, pore size of 2–12 nm, BET of 800 m^2^ g^−1^) was purchased from Nanjing XFNANO Materials Tech Co., Ltd. (China). Other reagents, such as DL-phenylalanine, methanol, acetone and toluene were purchased from Sinopharm Chemical Reagent Co., Ltd. (Shanghai, China).

### Enzyme production and purification

The recombinant *Escherichia coli* BL21 (DE3)/pET-28-*pal* [16] was used for the production of *Rg*PAL. The cells were grown to an OD_600_ of 0.6, and the enzyme expression was induced using IPTG (final concentration 0.4 mM). After incubation at 24°C for 20 h, the cells were collected by centrifugation (5 min, 4°C, 10000×g), washed twice with 50 mM sodium phosphate buffer (containing 10 mM imidazole, 150 mM NaCl, pH 7.5) and disrupted by sonication on ice at 40% power. After centrifugation, the supernatant was stored at 4°C. The enzymes were purified by His-tag-purification using an Akta-purifier (GE Healthcare). The proteins were loaded onto a HisTrap FF crude column (GE Healthcare), and the column was then washed using the same buffer and 58.3% of the elution-buffer (containing 250 mM imidazole, 150 mM NaCl). After elution, the enzyme was desalted using a HiPrep 26/10 desalting column (GE Healthcare) and equilibrated with 50 mM Tris-HCl buffer (pH 8.6). The purity of the sample was determined by SDS-PAGE.

### The adsorption of *Rg*PAL on MCM-41

An appropriate quantity of pure *Rg*PAL was dissolved in Tris-HCl buffer (10 mL, pH 8.6) by stirring in a conical flask at room temperature for a defined period. Then, MCM-41 (1 g) was added to the enzyme solution. The conical flask was maintained for 2 h at 30°C in an oscillator shaking at 150 rpm. The resulting absorbed enzymes were centrifuged and washed three times with distilled water. The absorbed *Rg*PAL derived from the above support is denoted MCM-41-*Rg*PAL. The enzyme loading amount (mg/g support) and activity recovery (%) were calculated according to the formulas S1 and S2 (Supporting information S1). The concentration of the enzyme protein was measured by the Bradford method [17].

### Chemical modification of MCM-41

The grafting of amino groups onto MCM-41 was accomplished through the methods of Jung et al. [18], Kannan et al. [19] and Zhao et al. [20] with minor modifications. The process was as follows: MCM-41 (1.0 g), toluene (50 mL) and 3-aminopropyltrimethoxysilane (2.0 mL) were added into a round bottom flask. The reaction mixture was refluxed under magnetic stirring at 180°C for 24 h. After cooling, the solid product was recovered by vacuum filtration and washed with toluene (100 mL) and acetone (100 mL) successively, then dried under vacuum for 12 h. The samples obtained were designated as MCM-41-NH_2_ (Fig. S1). The infrared spectroscopy (NICOLET NEXUS 470, Thermo Electron Corporation) was used to detect the amino groups on MCM-41 in the range of 4000 to 500 cm^−1^.

### Immobilization of *Rg*PAL on MCM-41-NH-GA

MCM-41-NH_2_ (1 g) reacted with 25 ml of glutaraldehyde (GA) (0.5% (v/v)) in Tris-HCl buffer (50 mM, pH 8.0) for 1 h at room temperature. The product was washed exhaustively with distilled water till excess GA was removed, and then dried at room temperature. The sample was designated as MCM-41-NH-GA (Fig. S1). Then, the *Rg*PAL was immobilized on MCM-41-NH-GA by mixing 1 g of MCM-41-NH-GA with 50 mg enzyme protein at room temperature for 2 h. The resulting complex was designated as MCM-41-NH-GA-*Rg*PAL (Fig. S1).

### Activity assay and biochemical characterizations of MCM-41-NH-GA-*Rg*PAL

The activity of both the free and MCM-41-NH-GA-*Rg*PAL was measured according to Zhu et al. [16]. The immobilization-specific activity and relative activity were assayed using the equations S3 and S4 (Supporting information S1).

The effects of pH on the activity were assayed at 50°C for 30 min using a series of buffers with various pH values (pH 4.0–7.0, 25 mM sodium acetate buffer; pH 7.0–9.0, 25 mM Tris-HCl buffer; pH 9.0–11.0, 25 mM sodium carbonate buffer). The effects of temperature on the activity were assayed at pH 8.5 for 30 min at various temperatures (30–65°C).

### Operational process of the recirculating packed-bed reactor (RPBR)

The resolution of DL-phenylalanine was performed in a RPBR (a borosilicate glass column, 25 cm length ×3.6 cm diameter, 450 mL, Shanghai Sangon Biotech Co., Ltd, China) (Fig. S2). The MCM-41-NH-GA-*Rg*PAL (100 U) and diatomite (100 g) were mixed and packed into the reactor. The diatomite was used to improve the filtration. The temperature was maintained at 50°C by circulating water. The reaction was initiated by pumping substrate solution (pH 8.5) through the column, and the reaction products were periodically recovered at the reactor outlet for analyses.

### Assay of D-phenylalanine and L-phenylalanine

The concentration of D-phenylalanine and L-phenylalanine were detected by HPLC on a C_18_ column (4.6 mm×75 mm, Hitachi, Japan) at 205 nm according to the method described by Fukuhara [21]. The mobile phase contained 20% (v/v) methanol and a complex of optically active L-Pro-Cu (II) (1.5 mM L-Pro and 0.75 mM CuSO_4_).

### Data analysis

The conversion ratio of L-phenylalanine to *trans*-cinnamic acid, the enantiomer excess value (*ee*_D_) of D-phenylalanine, the productivity of D-phenylalanine, the residence time (T_r_) and the volumetric conversion rate of L-phenylalanine were calculated by the equations S5–S9 (Supporting information S1).

## Results and Discussion

### Immobilization of *Rg*PAL by adsorption of MCM-41

**Figure 1:**
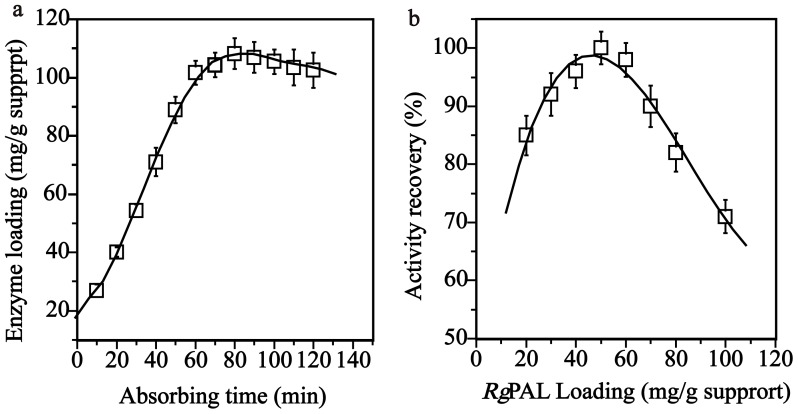
The effect of absorbing time on the enzyme loading amount (a) and effect of enzyme loading amount on the activity recovery (b).

Inorganic supports for enzyme immobilization are of great interest because of their durability, relatively low cost, and high mechanical strength for use in reactors. Among them, the mesoporous silica carriers with a high specific surface area and a large-pore volume are a promising family of materials for enzyme immobilization [22]. Mesoporous silica materials, such as MCM-41, have gathered significant attention in both academia and industry because of their large surface area and volume, tunable pore size and structures, openness to a wide variety of chemical modifications, and convenience of reutilization [20]. The pore diameters of the mesoporous silica materials are in the range of 1 to 30 nm, which is similar in size to enzyme molecules and suitable to load enzymes [23], [24]. In this study, we selected MCM-41 as immobilization supports. The pore size of MCM-41 is 2–12 nm, similar to the size of PAL (diameter of 9.5 nm) [25], which may facilitate the entry of *Rg*PAL. The BET (Brunauer-Emmett-Teller) surface area of MCM-41 reaches up to 800 m^2^ g^−1^, and this large surface area and high pore volume are suitable for immobilizing the enzyme [22]. To determine the absorption capacity of the carrier, 0.2 g enzyme was mixed with 1.0 g of MCM-41. The amount of loading enzyme was increased according to the incubation time with MCM-41, and reached a maximum (108 mg/g support) at 80 min (Fig. 1a). The absorption time was controlled and reached the highest at approximately 80 min. Subsequently, MCM-41 was mixed with 10–100 mg of enzyme to determine the optimal loading amounts. As shown in Fig. 1b, the activity recovery increased with the amount of loading enzyme, and the highest activity recovery was obtained with an amount of 50 mg enzyme/g support. The activity recovery decreased when the amount of loading enzyme exceeded 50 mg/g support, indicating that the excess enzyme may be embedded into the pore of MCM-41 and blocking the access of substrate and products.

### Effect of chemical modification of MCM-41 on immobilization

**Figure 2:**
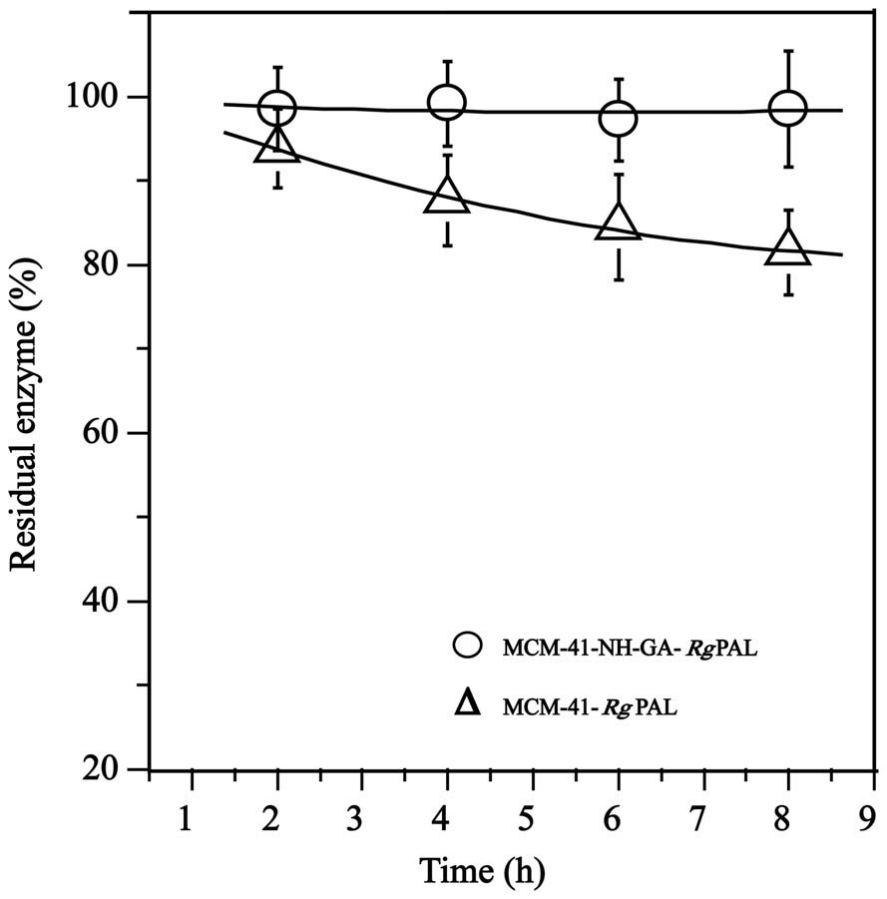
The effect of modification of support on enzyme binding. An amount of 0.1 g MCM-41-*Rg*PAL and MCM-41-NH-GA-*Rg*PAL were dissolved in 10 mL Tris-HCl buffer (25 mM, pH 8.6) and stirred in a conical flask at room temperature. The residual enzyme was subsequently determined every 2 h.

**Figure 3:**
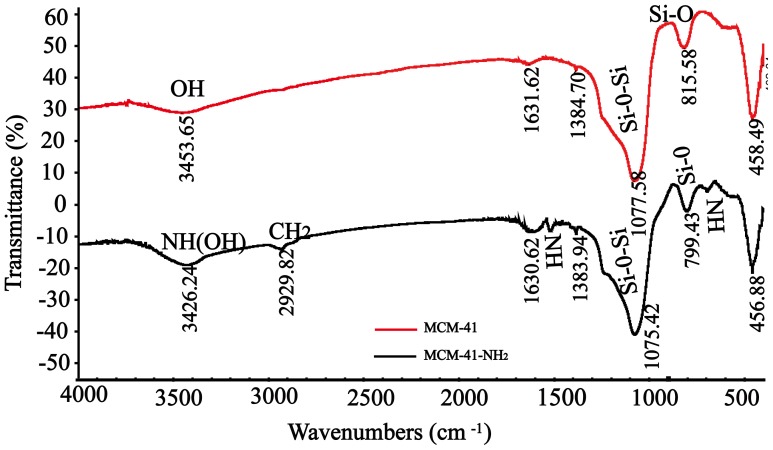
The FT-IR spectra of MCM-41 and MCM-41-NH_2_. The FTIR spectra were recorded using NICOLET NEXUS FTIR-470 (Thermo Electron Corporation) in the range of 4000–500 cm^−1^.

The interaction between MCM-41 and the enzyme is from weak physical forces, i.e., van der Waals or dispersion forces, and may be too weak to hold the enzymes such that *Rg*PAL may be prone to leaching during reaction and washing. As shown in Fig. 2, nearly 20% of the enzyme was leached from the MCM-41-*Rg*PAL within 8 h. Chemical modification of MCM-41 with proper organic groups can enhance the interaction of the enzyme with the support material and increase the operational stability of the immobilized enzyme, which may resolve the leaching problem. Silanol groups (Si-OH) on MCM-41 can serve as the sites for the anchoring of organic groups [19]. The amino of 3-aminopropyltrimethoxysilane (APTS) was grafted onto MCM-41 (Fig. S1). As shown in Fig. 3, a wide band at approximately 3,426 cm^−1^ was observed belonging to the stretching of a NH bond and the silanol groups (Si-OH). The band at 2,929 cm^−1^ is characteristic of the vibration of CH_2_ groups of the propyl chain of the silylating agent [19]. MCM-41 has a band at 1,075 cm^−1^ designating the vibration of Si-O-Si. The band at 815 cm^−1^ is attributed to the symmetrical and asymmetrical stretching of Si-O. The Si-OH vibration band decreases at approximately 815 cm^−1^ due to the interaction between NH groups [26]. The presence of bands at approximately 3,426 (NH vibrations), 1,555 (bending NH), and 692 cm^−1^ (bending NH) confirm the incorporation of amino groups. The presence of NH bending vibrations at 692 cm^−1^ and NH_2_ symmetric bending vibrations at 1555 cm^−1^ in MCM-41-NH_2_, absent in neat MCM-41, indicated that the amino was successfully grafted onto the MCM-41, resulting in MCM-41-NH_2_. After glutaraldehyde (GA) cross-linking, the immobilized enzyme MCM-41-NH_2_-GA-*Rg*PAL was obtained. As shown in Fig. 2, almost no enzyme was leached from the MCM-41-NH-GA-*Rg*PAL during the reaction.

### Effects of pH and temperature on the activity of MCM-41-NH-GA-*Rg*PAL

**Figure 4:**
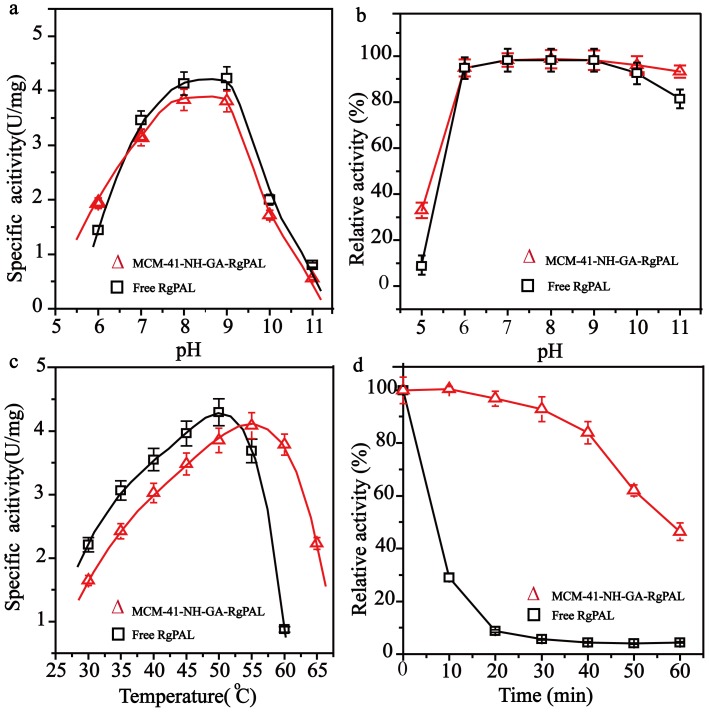
The characteristics of free *Rg* PAL and MCM-41-NH-GA-*Rg* PAL. (a) The optimal pH of free *Rg*PAL and MCM-41-NH-GA-*Rg*PAL. The effects of pH were determined at 50°C using a series of buffers with various pH values. (b) The pH stability of free and immobilized *Rg*PAL. The activity was assessed following the enzyme treatment in buffer at different pH values (5.0–12) for 12 h. The relative activity at pH 8.5 was defined as 100%. (c) The optimal temperature of free and MCM-41-NH-GA-*Rg*PAL. (d) The thermostability of free *Rg*PAL and MCM-41-NH-GA-*Rg*PAL. The free *Rg*PAL and MCM-41-NH-GA-*Rg*PAL were incubated at 60°C for 10–60 min. The relative activity before incubation was defined as 100%. The values presented correspond to the mean values of at least three replicates.

The effects of pH and temperature on MCM-41-NH-GA-*Rg*PAL were investigated. As shown in Fig. 4, the optimum pH of MCM-41-NH-GA-*Rg*PAL was between pH 8–9 (Fig. 4a), which was similar to the free enzyme. Moreover, the immobilized enzyme was more stable than free enzyme under acidic and alkaline conditions (i.e., lower than pH 6 or higher than pH 9) (Fig. 4b). The optimal temperature for MCM-41-NH-GA-*Rg*PAL activity was shifted to 55°C, compared to free enzyme at 50°C (Fig. 4c). MCM-41-NH-GA-*Rg*PAL exhibited a maximum specific activity of 4.08 U/mg at 55°C. In addition, MCM-41-NH-GA-*Rg*PAL was more stable than free enzyme at 60°C. The free enzyme had only 10% activity remaining after incubation at 60°C for 20 min, whereas the immobilized enzyme retained 95% activity (Fig. 4d). The enhanced thermal and pH stabilities observed for the MCM-41-NH-GA-*Rg*PAL are mainly due to the fact that *Rg*PAL molecules are covalently bonded on the silica surface, which offers good protection against pH and temperature alterations. Although MCM-41-NH-GA-*Rg*PAL was more stable than the free enzyme, the specific activity of MCM-41-NH-GA-*Rg*PAL was slightly lower than that of free enzyme. This may be due to the diffusion limitation of the substrate into the porosity of the support material and a lower degree of dispersion in the mesoporous matrix [27], or the deactivation of several enzyme active sites because of a perturbation in the conformation of the protein molecule bound to the silica surfaces [28].

### Effect of reusability on the activity of the MCM-41-NH-GA-*Rg*PAL

**Figure 5:**
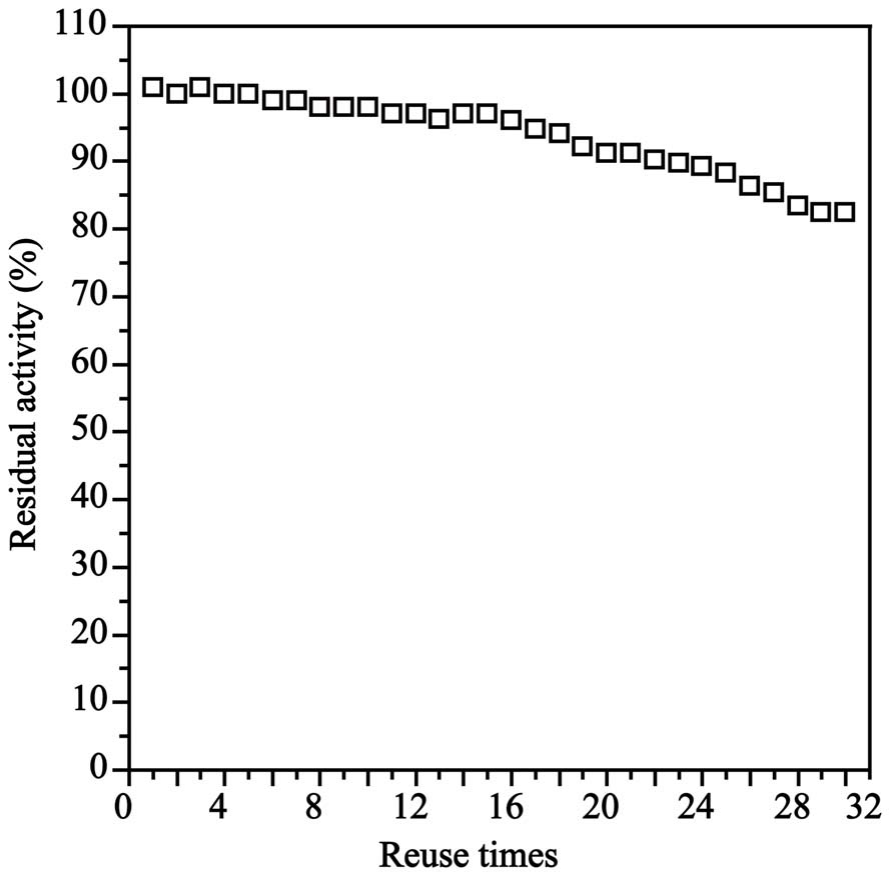
The reusability of MCM-41-NH-GA-*Rg* PAL. The initial activity of MCM-NH-GA-*Rg*PAL was measured and then compared with the activity of the used enzyme obtained after its repeated use per day. After each cycle, MCM-41-NH-GA-*Rg*PAL was immediately filtered, washed with buffer solution and stored at 4°C.

One of the attractive advantages of immobilized enzymes for large scale industrial applications is that the immobilized enzyme can be easily separated from the reaction system and allowed to perform repeatedly, which would significantly lower the cost of the production process. MCM-41-NH-GA-*Rg*PAL was collected from the reaction mixture by centrifugation, then washed and reused. As shown in Fig. 5, up to 80% activity of MCM-41-NH-GA-*Rg*PAL was retained after 30 reuses. These results suggest that MCM-41-NH-GA-*Rg*PAL is stable and suitable for industrial application.

### Resolution of DL-phenylalanine using MCM-41-NH-GA-*Rg*PAL

**Figure 6:**
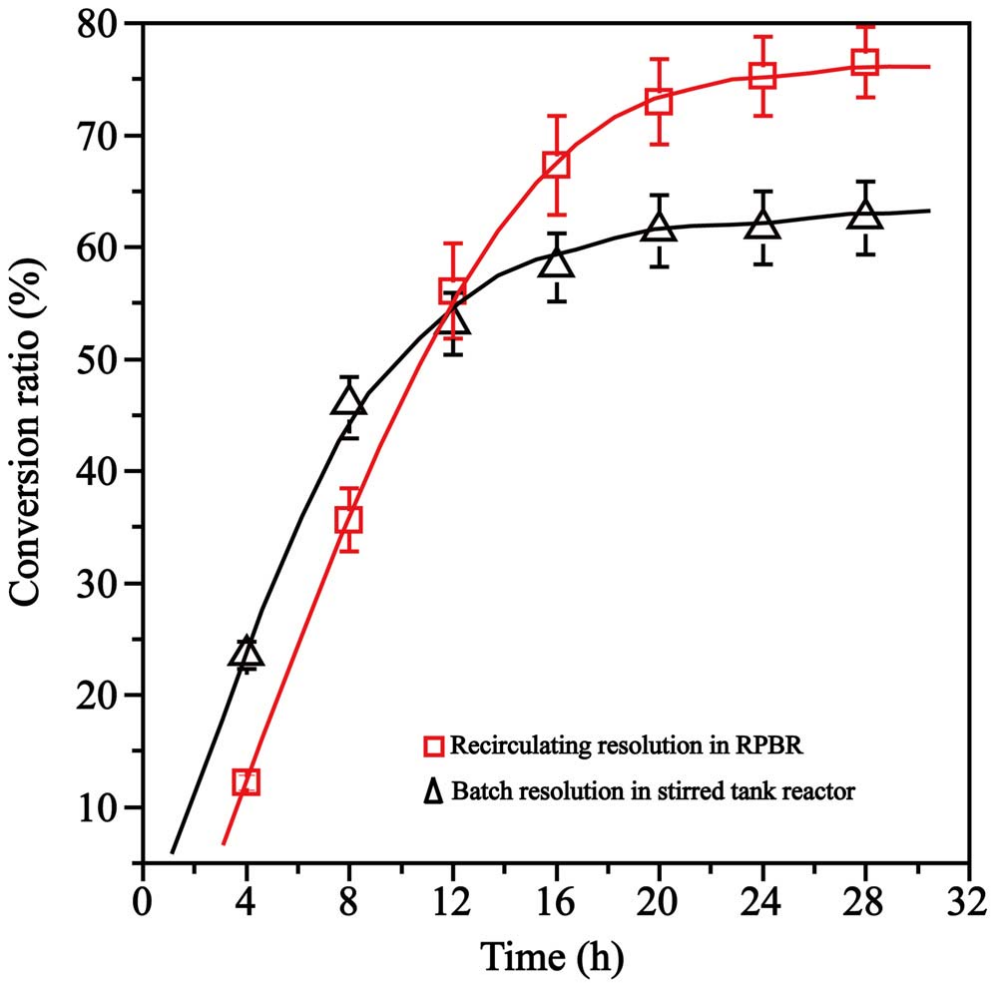
Comparison of resolution of DL-phenylalanine in a RPBR and in a batch stirred-tank reactor. Two equal amounts of MCM-41-NH-GA-*Rg*PAL (100 U) were loaded in a RPBR and a batch stirred-tank reactor, respectively. The substrate of 1 liter (100 mM) were fed to the RPBR with a flow rate of 8 mL min^−1^, or directly added in the batch stirred-tank reactor. The reaction products were periodically collected at the reactor outlet and analyzed for the conversion ratio.

The resolution efficiency of DL-phenylalanine using immobilized MCM-41-NH-GA-*Rg*PAL was tested in a RPBR and a stirred-tank reactor. The substrate (100 mM, 1 L) was fed into a RPBR with a flow rate of 8 mL min^−1^ and directly added into a stirred-tank reactor (stirred glass reactor, 4 L). As shown in Fig. 6, the conversion ratio of L-phenylalanine in the batch stirred-tank reactor was higher than that in the RPBR in 12 hours, which may be due to the increase association of the enzyme and the substrate in the stirred-tank reactor. However, the conversion ratio remained at approximately 60% after 16 h. The L-phenylalanine was not able to be converted completely, because the enzymatic activity was significantly inhibited by the *trans*-cinnamic acid product and D-phenylalanine. Compared to the reaction in the bath stirred-tank reactor, the inhibition was significantly relieved by using the recirculating process in a RPBR, and the conversion ratio was significantly increased (Fig. 6). The RPBR is more suitable than a batch stirred-tank reactor when the enzyme is subject to product inhibition, and the inhibition was significantly relieved because the residence time (the retention time of the substrate with the enzyme) may be small enough to avoid the product inhibition [29]. Moreover, the RPBR showed many advantages over the batch stirred-tank reactor: the immobilized enzyme could be reused without a prior separation; the ratio of enzyme to substrate was much lower than that in the stirred-tank reactor; the enzyme loss was reduced by the absence of mechanical shear stress, resulting in long-term stability and high reaction performance, etc. [30], [31].

### Optimization of the operational process of the RPBR

**Figure 7:**
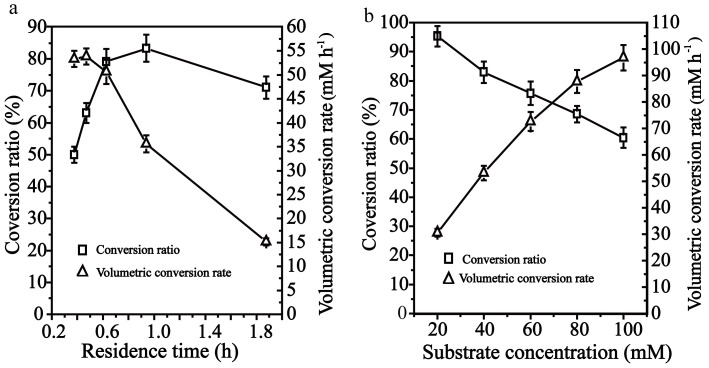
Effects of residence time and substrate concentration on the resolution of DL-phenylalanine. To compare the conversion ratio and volumetric conversion rate under different operational conditions, the reactor was run for 12 h, and then the sample was obtained from the reactor analysis. (a) The effect of residence time on the conversion ratio and the volumetric conversion rate. The substrate (40 mM, 1 L) was fed into the RPBR with different flow rates to provide different residence times. (b) The effect of substrate concentration on the conversion ratio and volumetric conversion rate. The substrate (1 L) was fed into a RPBR with a flow rate 12 mL min^−1^.

The influences of residence time and substrate concentration on the resolution of DL-phenylalanine in a RPBR were further investigated. The residence time indicates the retention time of the substrate mixture in the reactor with the enzyme. Thus, residence time is dependent on the flow rate of the substrate and the dimensions of the packed-bed reactor. In this study, residence time was controlled by the pumping speed of the substrate. As shown in Fig. 7, the conversion ratio was increased with the increase of residence time, and reached the maximum level at 0.93 h (flow rate of 8 mM/min). However, the volumetric conversion rate decreased as residence time increase (Fig. 7a). The low residence time (high flow rate) improves mass transfer of the reactants from the bulk of the reactant mixture to the enzyme surface and thus increases the reaction rate [20]. Further decrease of residence time may result in the large decrease in the conversion ratio, which may due to the decrease in the contact time of the substrate and the immobilized biocatalyst. Therefore, the optimal residence time was determined as 0.625 h (flow rate of 12 mM/min) to reach a high conversion ratio and volumetric conversion rate simultaneously.

**Figure 8:**
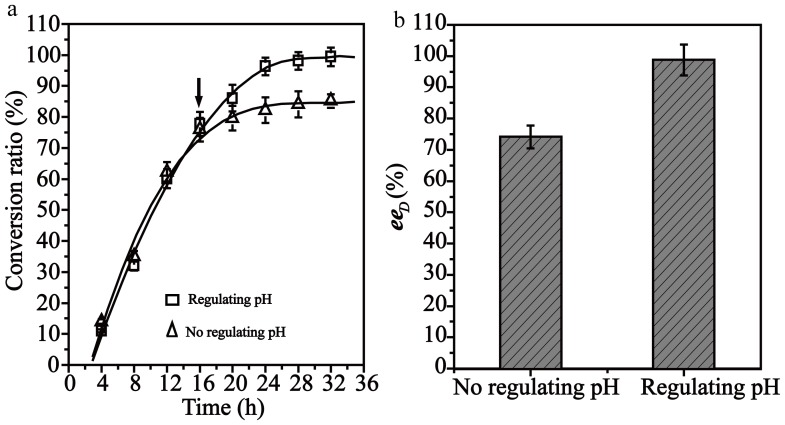
The effect of controlling pH on the resolution. (a) Effects of pH regulation on the conversion ratio. (b) Effects of pH regulation on *ee*_D_. The substrate (100 mL, 1 L) was added to the reactor with a flow rate of 12 mL min^−1^. When the resolution was performed for 16 h, the pH was adjusted from 8.5 to approximately 5 to precipitate the *trans*-cinnamic acid from the reaction solution. The *trans*-cinnamic acid was removed by filtration, and then the pH of the reaction was readjusted to 8.5, and the resolution was continued.

The effects of DL-phenylalanine concentration on resolution were investigated. As shown in Fig. 7b, the volumetric conversion rate increased as the concentration of substrate increased, and reached 96.7 mM h^−1^ by feeding 100 mM substrate (the saturated solubility of substrate) with a residence time of 0.625 h (flow rate of 12 mL/min). The conversion efficiency in a RPBR could be increased by increasing the substrate feeding concentration until the catalytic ability became the limiting factor [32]. The resolution efficiency in the RPBR was dependent on the volumetric conversion rate. Although the resolution efficiency in the RPBR increased by increasing the substrate feeding concentration, the conversion ratio decreased with the increase in substrate concentration, which may result from the inhibition of the *trans*-cinnamic acid product and D-phenylalanine. The conversion ratio remained at approximately 80% after 20 h, and the L-phenylalanine was unable to be completely converted (Fig. 8a). The solubility of the *trans*-cinnamic acid is low at acidic pH. Therefore, the inhibitory effect was relieved by separating the *trans*-cinnamic acid through pH adjustment. At 16 h, the pH was adjusted from 8.5 to approximately 5 to precipitate the *trans*-cinnamic acid form the reaction solution. The *trans*-cinnamic acid was removed by filtration, and the resolution operation continued after the reaction system pH was readjusted to 8.5. As shown in Fig. 8a and 8b, both the conversion ratio and *ee*_D_ exceeded 99% at 28 h, and the productivity of D-phenylalanine achieved 0.34 g L^−1^h^−1^.

### The operational stability of the RPBR

**Figure 9:**
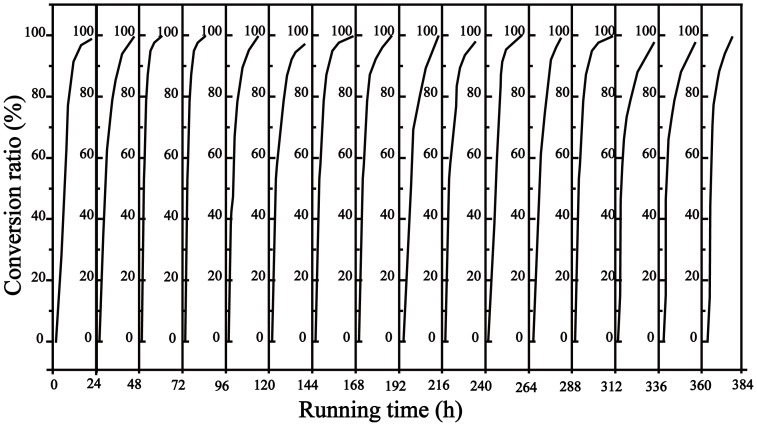
Operation stability of the RPBR. For each batch, 100 mM of substrate was recycled through the RPBR with a flow rate 12 mL/min. At 16 h, the pH was readjusted from 8.5 to approximately 5 to precipitate the *trans*-cinnamic acid. After the *trans*-cinnamic acid was removed by filtration, the pH of the reaction was readjusted to 8.5 to continue, and each batch run for 24 h. The reaction solution was sampled for analysis every 4 h.

The operational stability of a system is an important parameter in an industrial process, because it directly affects the costs. The operational stability of the reactor was studied for a prolonged period under the optimized conditions. The RPBR was operated for 16 batches for a total of 384 h under the optimal conditions to evaluate the operational stability. For each batch of the reaction, 100 mM DL-phenylalanine (1 L) was recycled through the RPBR with a feeding rate of 12 mL h^−1^ (residence time of 0.625 h), and the *trans*-cinnamic acid was precipitated by pH adjustment after a reaction time of 16 hours. As shown in Fig. 9, the conversion ratio still remained above 95% after operating for16 batches in the RPBR, suggesting that the RPBR is usable for a large scale application.

### The performance of the scaled-up RPBR

**Figure 10:**
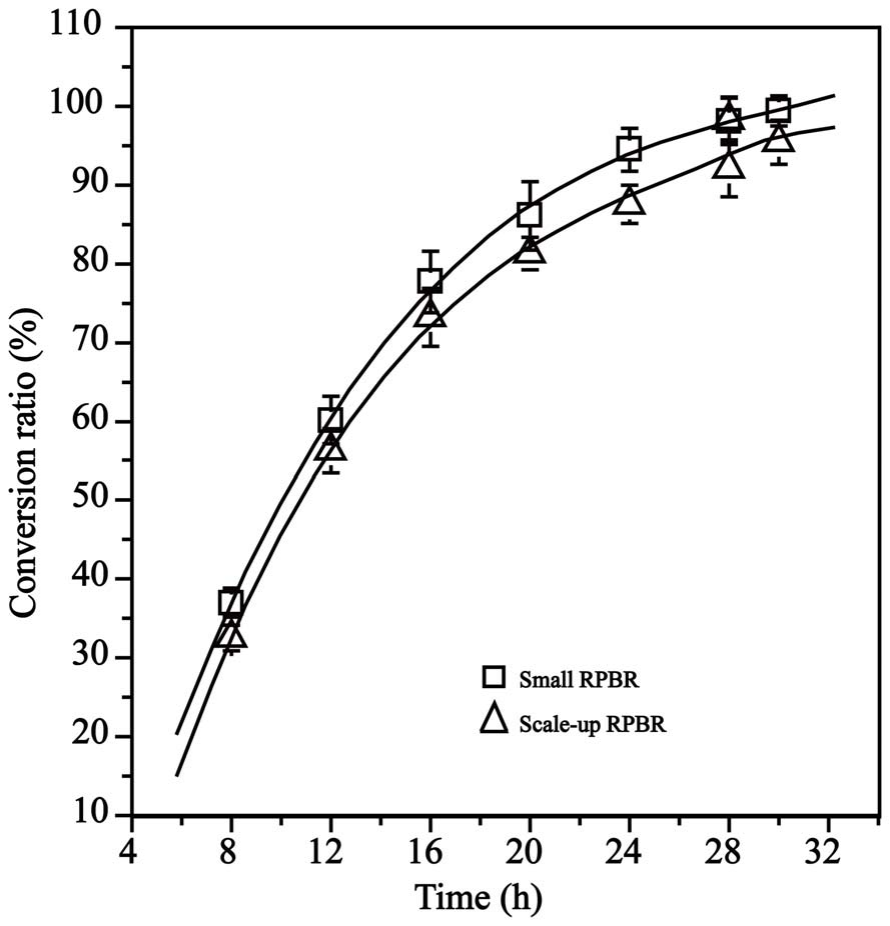
Resolution curves of DL-phenylalanine in the small RPBR and scaled-up RPBR. The volume of the reaction solution and the quantity of enzyme used were increased proportionally with the reactor size. The operation process in the scaled-up reactor maintained a similar residence time (0.625 h) and load of substrate (25 L of 100 mM), and the pH was regulated from 8.5 to approximately 5 to precipitate the *trans*-cinnamic acid at 16 h. After the *trans*-cinnamic acid was removed by filtration, the pH of the reaction was readjusted to 8.5, and the reaction was continued.

To investigate the feasibility of industrial processes, the RPBR was scaled up 25-fold. The volume of the reaction solution and the quantity of the immobilized enzyme used increased proportionally with the reactor size. The operation process maintained a similar residence time (0.625 h) for the small-scale reactor, and *trans*-cinnamic acid was precipitated from the reaction solution by pH adjustment at 16 h. The performance of the immobilized enzyme in the scaled-up reactor was comparable with the small-scale packed-bed reactor. The resolution curves of the reactors can be nearly superimposed on those from the small-scale reactor (Fig. 10). The productivity of D-phenylalanine (*ee*_D_>99%) reached 7.2 g L^−1^h^−1^. The conversion ratio of the resolution in the scale-up was approximately 5% lower than that in the small scale reactor. Compared with the small scale reactor, it is more difficult to control flow rate or residence time in the scaled-up reactor than in the small reactor, and the mass transfer limitation in the scaled-up reaction system may be greater than that in the small reactor, which may reduce the intrinsic enzyme activity [33]. Therefore, further optimization is needed to improve the resolution efficiency in the scaled-up reactor, and such studies are being performed.

According to these results, this process for D-phenylalanine production showed advantages to the existing hydantoinase-carbamoylase process in the following aspects. Firstly, the feedstock DL-phenylalanine is available and commercially produced at low cost by fermentation and chemical synthesis [12]. In contrast, the hydantoinase–carbamoylase process requires 5-substituted hydantoin as feedstock which is synthesized using highly toxic potassium or sodium cyanide according to Bucherer-Berg method [34]. Secondly, the hydantoinase-carbamoylase process is a multi-enzyme reaction system involving three enzymes (hydantoinase, carbamoylase and racemase), and the reaction conditions of the three enzymes are different. Obviously, the production of three enzymes and the multi-enzyme catalytic process control are more complicated than a single *Rg*PAL reaction. Thirdly, the stability of N-carbamoylase is found to be low compared to that of D-hydantoinase, which is considered one of the limiting factors in the process [35]. The immobilized *Rg*PAL showed high stabilities and 80% activity of was retained after 30 reuses. In addition, the immobilized *Rg*PAL in RPBR showed excellent operational stability, the RPBR was continuously operated for 16 batches for a total of 384 h, the conversion rate didn't decrease, which could remarkably reduce the production cost. Other limited exploitation of hydantoinase-carbamoylase process in industry could be listed as narrow range of substrate catalysis offered by known hydantoinase enzymes and slow racemization rates [36]. The asymmetric resolution using immobilized *Rg*PAL in RPBR might be an alternative method.

## Conclusions

An efficient process was developed to produce D-phenylalanine through resolution of DL-phenylalanine using immobilized *Rg*PAL in a recirculating packed-bed reactor. Under optimal operational conditions, the conversion ratio and volumetric conversion rate of L-phenylalanine were 99% and 96.7 mM h^−1^, respectively, and the maximum productivity of D-phenylalanine (*ee_D_*>99%) reached 0.32 g L^−1^h^−1^. The resolution process was further scaled up 25-fold, and the maximum productivity of D-phenylalanine (*ee_D_*>99%) was obtained with 7.2 g L^−1^h^−1^. To our knowledge, this is the first report on the D-phenylalanine production through asymmetric resolution of racemic DL-phenylalanine using immobilized *Rg*PAL.

## Supporting Information

Supporting Information S1 **Equations used in this study.**
(DOCX)
