# Supplementary material for: Efficient Preparation of Enantiopure D-Phenylalanine through Asymmetric Resolution Using Immobilized Phenylalanine Ammonia-Lyase from Rhodotorula glutinis JN-1 in a Recirculating Packed-Bed Reactor
Source: PLoS One. 2014 Sep 30;9(9):e108586. doi: 10.1371/journal.pone.0108586 (PMC4182499; doi:10.1371/journal.pone.0108586)
Supplement: Supporting Information S1 — Equations used in this study. (DOCX) [file pone.0108586.s001.docx]

**Supplementary material**

**Efficient preparation of enantiopure D-phenylalanine through asymmetric resolution using immobilized phenylalanine ammonia-lyase from *Rhodotorula glutini* JN-1 in a recirculating packed-bed reactor**

Longbao Zhu ^a,b,^*, Li Zhou ^a,^*, Nan Huang ^a^, Wenjing Cui ^a^, Zhongmei Liu ^a^, Ke Xiao ^a^, Zhemin Zhou ^a,¶^

^a^ Key Laboratory of Industrial Biotechnology, Ministry of Education, School of Biotechnology, Jiangnan University, Wuxi 214122, Peoples Republic of China.

^b^ School of Biochemical Engineering, Anhui Polytechnic University, 8 Zheshan Road, Wuhu, Anhui 241000, Peoples Republic of China.

* These authors contributed equally to this work.

¶ To whom correspondence should be addressed.

Zhemin Zhou, Tel: +86-510-85325210, Fax: +86-510-85197551,

E-mail: [zhmzhou@jiangnan.edu.cn](mailto:zhmzhou@jiangnan.edu.cn)

**Supporting information S1**

 (S1)

 (S2)

 (S3)

 (S4)

 (S5)

 (S6)

 (S7)

 (S8)

 (S9)

Where, M_pal, in_ is the total protein content added to the mixture (mg); M_pal, out_ is the protein content

of the washing solution and supernatant after immobilization (mg); M_support_ is the weight of the support (g); L_phe, in_ is the concentration of L-phenylalanine in the inlet solution (mM); L_phe, out_ is the oncentration of L-phenylalanine in the outlet solution (mM); D_phe, out_ is the concentration of D-phenylalanine in the outlet solution (mM); h is the time when the *ee*_D_ exceeds 99 %; F is the flow rate (mL/min) of the substrate, V is the reactor volume (mL).

**Supplementary figure legends**

**Figure S1. Schematic illustration of the covalent methods of immobilized *Rg*PAL on an MCM-41 support.**

The amino group was grafted onto MCM-41 to generate MCM-41-NH_2_, and then the bifunctional glutardealdehyde (GA) was used to cross link the enzyme and MCM-41-NH_2_ to generate MCM-41-NH-GA-*Rg*PAL.

**Figure S2. Schematic diagram of the resolution of DL-phenylalanine using immobilized *Rg*PAL in a RPBR.**

The immobilized *Rg*PAL was packed into a RPBR. Substrates were fed from the bottom of the column using a controlled peristaltic pump. The solution was recirculated until the reaction nearly reached completion. The products were collected in a separate tank. Samples of the input and output were collected and analyzed by HPLC.
